# A call for individualized evacuation strategies for floods: A case report of secondary surgical site infection in a postsurgery breast cancer patient in Fukushima, Japan, following Typhoon Hagibis in 2019

**DOI:** 10.1002/ccr3.3727

**Published:** 2021-01-15

**Authors:** Akihiko Ozaki, Yoshiaki Kanemoto, Masahiro Wada, Tomohiro Kurokawa, Ayumu Kawamoto, Toyoaki Sawano, Divya Bhandari, Masaharu Tsubokura, Tetsuya Tanimoto, Tomozo Ejiri, Norio Kanzaki

**Affiliations:** ^1^ Department of Breast Surgery Jyoban Hospital of Tokiwa Foundation Iwaki Fukushima Japan; ^2^ Department of Surgery Jyoban Hospital of Tokiwa Foundation Iwaki Fukushima Japan; ^3^ Department of Breast Surgery Sano Kosei General Hospital Sano, Tochigi Japan; ^4^ Faculty of Medicine University of Szeged Szeged Hungary; ^5^ Department of Surgery Sendai City Medical Center Sendai Open Hospital Sendai Japan; ^6^ Department of Radiation Health Management Fukushima Medical University School of Medicine Iwaki Fukushima Japan; ^7^ Community and Global Health Graduate School of Medicine the University of Tokyo Tokyo Japan; ^8^ Department of Internal Medicine Jyoban Hospital of Tokiwa Foundation Iwaki Fukushima Japan

**Keywords:** breast neoplasms, climate change, cyclonic storms, floods, Japan

## Abstract

Recognition of Individual and environmental risks is crucial to alleviate damage inflicted by disasters. In particular, an awareness of floods and their health risks in patients’ residences is important for patients and their healthcare professionals.

## Graphical abstract

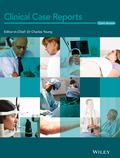

## 1 INTRODUCTION

Cancer patients, particularly those postsurgery, are prone to skin infection following immersion in flood waters. We reported secondary surgical site infection in a postoperative 80‐year‐old postoperation breast cancer patient affected by Typhoon Hagibis. Individualized responses based on individual and environmental risks are crucial to alleviate the damage of disasters.

Flood frequency has been increasing globally due to climate change.^1^ When flood water submerges residential districts, it is affected by sewage and can easily cause skin infections in the affected population.^2^ When considering populations vulnerable to skin infections following flooding, the risk may be higher in postsurgery cancer patients than the general population because the immune systems of cancer patients are suppressed^3^ and they are particularly compromised after chemotherapy, radiotherapy, or surgery.^2^ Incisional wounds are especially susceptible to infection. However, little information is available concerning measures to prevent such infections among these populations following floods.

Typhoon Hagibis, which occurred on 6 October 2019 and landed on Japan's main island on 12 October 2019, killed approximately one hundred citizens and caused substantial other injuries.^4^ These damages occurred although typhoon warnings had been repeatedly disseminated in various media outlets beforehand. This typhoon was a Category 5 (the highest according to the Saffir‐Simpson Hurricane Wind Scale) and was one of few severe disasters that required government support in its recovery. Given this context, we present a postoperative breast cancer patient who was affected by flood water caused by Typhoon Hagibis in order to plan specific measures to ensure the safety of cancer patients from flood damage in the future.

## 2 CASE DESCRIPTION

An 80‐year‐old patient living with her son‐in‐law and grandson in Iwaki City, Fukushima, with chronic renal diseases of unknown cause, found a lump on the top of her right nipple and initially visited her primary physician in March 2019. She was then referred to our breast surgery outpatient office on 17 June 2019 after several consultation cancellations. An in‐depth diagnostic workup confirmed cancer of the right breast with anatomical clinical stage of cT2N0M0 StageⅡA. On 25 September 2019, we carried out mastectomy and sentinel lymph node biopsy which revealed positive lymph node metastasis. A subsequent axillary resection was performed on the same day. The patient's postoperative condition was stable, and she was discharged on 11 October 2019, the day before Typhoon Hagibis hit Iwaki City (postoperative day 16). Although she was advised to stay in the hospital until the typhoon passed by the city, she insisted on discharge that day.

At midnight on 12 October 2019, the typhoon hit the city with force, destroying 5927 residences and 3111 nonresidential buildings. 5721 buildings were submerged and 12 people were killed. The patient's single‐story residence was located 13 meters above sea level and only 100 meters away from the embarkments running along the Natsui River. The flood hazard map that estimates areas that would be submerged by floods with a scale of once in one thousand years is freely available on the municipality website and clearly showed that the patient's residence would become submerged under 5 to 10 meters of water due to overflow of the nearby Natui River. The typhoon actually destroyed several of the river's embarkments at 12:30AM on 13 October 2019. While Iwaki City issued an evacuation order for those living around the river at 9:40PM on 12 October 2019, she reportedly did not evacuate from her residence because she was not aware of that warning. The flood water submerged her residence soon after the destruction of embarkments while she was asleep. The water level rapidly reached approximately two meters high, and she managed to survive by climbing on furniture her son‐in‐law piled up for her. Despite efforts to cover her wound, it was immersed in contaminated flood water. The water smelled rotten, probably because of sewage that overflowed from the sewers, given that scattered manhole covers were observed around her residence the next day.

On 13 October 2019, she complained of appetite loss and visited our emergency department the next day (14 October 2019). On physical examination, she was afebrile, and her surgical site was not apparently infected, but she was hospitalized due to appetite loss and destruction of her residence. After admission, her surgical site gradually became red, swollen, and edematous, highly suggestive of secondary infection. On October 16, we inserted a drainage tube into the surgical wound and started administration of cefazolin 1g three times per day. We failed to take a culture of the fluid sample. Further discussion revealed that she had not carefully read the flood hazard map before the typhoon. Her condition gradually improved, and she was discharged on 5 November 2019. After discharge, she abandoned her original residence because of persistent smells and moved to a nearby apartment building following several relocations.

## 3 DISCUSSION

This case describes a skin infection in a postoperative breast cancer patient due to an immersion of the surgical site wound in contaminated flood water following Typhoon Hagibis. Her discharge took place on the day before the typhoon hit Iwaki City, and she had characteristics that would make her vulnerable to floods, such as advanced age, breast cancer, and postoperative status. In this regard, we believe that there might have been viable interventions to prevent her from incurring such severe flood damage.

Of note, a flood hazard map covering areas that would be submerged in floods was freely and widely available in Iwaki City. In Japan, local municipalities usually publish flood hazard maps on their websites and on paper for community distribution. Further, the Geospatial Information Authority in Japan, as similarly introduced by other organizations such as the Federal Emergency Management Agency in the United States and Ministry of Environment in France, prepares a website called “*Kasaneru (Superimposing) Hazard Map* [in Japanese]” where people can freely evaluate risks of flooding and landslides in basically any location in Japan. According to a municipality‐based hazard map, her neighborhood was designated as a flood‐prone area, while the hospital was not. Following Typhoon Hagibis, the patient's neighbors were all affected by flooding, but the hospital only incurred minor ceiling water leakage.

If healthcare professionals had understood the information from the flood hazard map beforehand, we could have given her more appropriate advice when she wished to be discharged the day before the typhoon hit the city. Specifically, we could have more strongly persuaded her to stay at the hospital to prevent potential harm. Further, even when she declined our suggestion, we could have alternatively advised her to evacuate to residences of family members or friends living outside flood‐prone areas. Needless to say, some medical institutions exist in flood‐prone areas, and so this approach may not always be generalizable.

For all residents, including cancer patients, as is a lesson from this case, it is important to know whether their residences are located in flood‐prone areas in ordinary times, but this may not be enough to guarantee a safe evacuation in situations when flood damage is anticipated. People are easily affected by normalcy bias, which is a tendency to consider that they themselves will not be affected by disasters or crises.^5^, ^6^ In this regard, it is unclear whether people can successfully evacuate just because they were aware of the warning in a timely fashion. Rather, they may have been affected by normalcy bias, and somehow considered that they would not be affected by the flood. It is not always easy to overcome normalcy bias, but informal and formal support and ordinary‐time preparedness would help people avoid this inherent mental tendency.^6^

Every natural disaster, including, but not only flooding, is unique. In this respect, lessons gained from one disaster are not directly applicable to another. For instance, dozens of people died in floodwater in Kumamoto, Japan in July 2020. The damage was not caused by typhoons, but by a seasonal rain front, and it was obviously more difficult to predict the occurrence, timing, and potential location than for typhoons. Thus, flexible responses accounting for actual disaster circumstances, human and institutional capacities, and patients’ conditions are needed. In this regard, our ongoing mission is a continuous accumulation of evidence on any kind of health issue related to flooding and other disasters to help safeguard the health and well‐being of cancer patients and other citizens when faced with disasters.

In conclusion, we reported a case of secondary surgical site infection in a postoperative breast cancer patient affected by Typhoon Hagibis. Individualized responses based on individual and environmental risks are crucial to alleviate damage from disasters. In particular, an awareness of flood risks in patients’ residences can be crucial for patients and their healthcare professionals.

## CONFLICT OF INTEREST

Akihiko Ozaki receives personal fees from MNES Inc, outside the submitted work. Also, Tetuya Tanimoto receives personal fees from MNES Inc, and Bionics co., ltd. outside the submitted work.

## AUTHOR CONTRIBUTIONS

AO: wrote the manuscript. All authors conceptualized and designed the case report and revised the paper. All authors read and approved the final manuscript.

## ETHICAL APPROVAL

Written informed consent was obtained from the patient for publication of this case report and any accompanying images.

## Data Availability

The patient has not agreed that her data are transferred to those involved in this project, and thus data related to this case report is not available to journal readers.
